# Effectiveness and safety of different medicines for Uremia pruritus: A protocol for systematic review and network meta analysis

**DOI:** 10.1097/MD.0000000000023043

**Published:** 2020-12-18

**Authors:** Shifan Yan, Ting Yu, Fusheng Li, Yong Huang, Lu Xiao, Haiqun Li, Maohong Wang

**Affiliations:** a Jiangxi University of Traditional Chinese Medicine; b Affiliated Hospital of Jiangxi University of Traditional Chinese Medicine, Nanchang, China.

**Keywords:** Uremia pruritus, network meta-analysis, protocol

## Abstract

**Introduction:**

A large number of patients will experience pruritus after uremia. Medicine is the preferred treatment for many doctors, but the effectiveness and safety of different medicines for uremia pruritus has not yet been comprehensively compared, based on network meta-analysis.

**Methods and analysis:**

According to the retrieval strategy, two team members independently searched the literature in 7 databases, and imported the retrieval results into the EndNote Software AQ8 (V.X9). After deleting repeated articles, they read the abstract and the full text, selected the articles that met the inclusion criteria and extracted valid information. The main results were visual analogue scale (VAS) and the secondary results were verbal rating scale (VRS), Dirk R Kuypers score, and adverse event incidence. The methodological quality evaluation was conducted from 7 aspects, according to The Cochrane Collaborative Tool, Stata Statistical Software (Version 14.0, Stata Corporation, College Station, TX) was used for data analysis. The level of evidence will be assessed by the Grading of Recommendations, Development and Evaluation (GRADE) instrument).

**Results:**

The results will rank the efficacy of drugs used to treat uremic pruritus and assess their safety.

**Conclusion:**

This study is the first to compare the efficacy and safety of medicines for uremic pruritus based on network analysis and will provide evidence and ideas for the treatment of uremic pruritus.

**INPLASY registration number:**

No. INPLASY202090103.

## 1 Introduction

Uremia pruritus is one of the most common complications in patients with renal disease at the end stage. Its clinical manifestations are bilateral symmetric skin pruritus, but the location is not fixed, which is more common in the face, back, and arms, At the same time, it can be combined with other symptoms such as papules, ulcers, and erosion.^[1–4]^ There is no consensus on the pathogenesis of uremic pruritus at present. Based on the current literature, it is found that uremic pruritus is mainly related to toxin accumulation,^[4]^ immune system disorder,^[5]^ peripheral neuropathy,^[6]^ and opioid disorder.^[7]^ At present, the most comprehensive epidemiological investigation is DOPPS,^[4]^ which conducted a study on adults with end-stage renal disease undergoing hemodialysis in many countries, and the results showed that about 37% of patients had at least moderate or above pruritus. The prevalence also varies by region, with 48% in the United Kingdom,^[4]^ 44% in Japan,^[8]^ 30% in the United States,^[9]^ and 26% in Germany,^[4]^ with at least moderate itching.

Although the clinical manifestations and incidence of uremic pruritus vary, studies have found that it is closely related to mood, social relations, and sleep, and problems such as decreased QoL and depression cannot be ignored.^[10–12]^ Moreover, uremic pruritus has been shown to be an independent predictor of mortality.^[13]^ There is no doubt that improving the quality of life of uremic pruritus patients has become a priority in renal disease research.^[14]^

The treatment of uremic pruritus mainly include drug therapy and alternative therapies, commonly used drugs have Anticonvulsants, Antihistamines, Antiemetic, Opioid agonist and antagonist, Immunosuppressant, Antidepressants, Mast cell stabilizer, etc.^[15]^ The efficacy of different drug is inconsistent, traditional meta analysis has limitations, because can only compare the curative effect between the two kinds of drugs, such as an article^[16]^ compared the meta analysis of gabapentin, and network meta-analysis can simultaneously comparing direct and indirect comparison of a variety of drugs, and the effect of sorting, find the most appropriate treatment.

## 2 Methods and analyses

### 2.1 Design

Systematic review and network meta-analysis.

### 2.2 Patient and public involvement

This study is a secondary literature study and does not involve clinical patients or the general public.

### 2.3 Eligibility criteria

#### 2.3.1 Types of studies

High-quality methodological articles are critical to the credibility of the results, so we included only RCTs with a variety of drugs for urinalysis. Since all patients with end-stage uremia are undergoing hemodialysis, there is no requirement for hemodialysis mode and flux. On the basis of hemodialysis, combined with various drugs, drugs are only limited to Gabapentin, Pregabalin, Tacrolimus, and Ondancetron. The intervention measures of the control group should include placebo or blank control.

#### 2.3.2 Type of participant

All adult patients diagnosed with uremic pruritus, the diagnostic criteria include uremic and pruritus and exclude other causes of pruritus, such as skin diseases, mosquito bites, etc.

#### 2.3.3 Interventions

The intervention measures were combined with a drug for uremic pruritus that was restricted to Gabapentin, Pregabalin, Tacrolimus, and Ondancetron. In addition, blank control and placebo were also included. There was no restriction on basic treatment between the test and control groups, but there was no difference between the two groups. If there are multiple groups and two of them meet the above requirements, they should also be included in the study.

#### 2.3.4 Types of outcome measurements

##### 2.3.4.1 Primary outcome

The visual analog scale (VAS)^[17]^: The VAS score was originally used to assess pain intensity, but is now also used to describe itch severity. It USES a line segment of 10 cm, with 0 representing no symptoms and 10 representing the highest intensity of itching, so that the patient can draw his or her own itching degree on the line segment. Itch severity was scored on a scale of 0 to 10. VRS included four grades of itch severity: none, low, medium, and severe.

##### 2.3.4.2 Secondary outcomes

1. The numeric Rating Scale (NRS)^[17]^: Divided the itch into four grades: no, low, moderate and severe.
2. The Dermatology QOL Index (DLQI)^[18]^: It includes 10 questions for assessing. The impact of itching on quality of life in terms of symptoms, job, and interpersonal relationship.
3. The incidence rate of adverse events.

#### 2.3.5 Exclusion criteria

1. Exclusion of reviews, animal experiments, case reports, and non-randomized controlled trials.
2. Exclude the test of administration method and dose of the study drug.
3. Exclude tests comparing different hemodialysis pathways.
4. Exclude tests with missing data.

### 2.4 Literature search

Two trained team members (YT and YSF) followed the retrieval strategy in seven databases (PubMed, Cochrane Library, Embase, Web of Science, Chinese National Knowledge Infrastructure (CNKI), Chinese Biomedical Literature Database (CBM), and Wanfang Database (WF) were used to comprehensively retrieve documents that met the requirements, and the retrieval strategy was reached through discussion by the panel members. The retrieval strategy of PubMed is shown in Table 1. Two team members independently searched the article according to the retrieval strategy, they also exported the citations.

**Table 1:** Search strategy used in PubMed database.

Number	Search items
#1	randomized controlled trial [pt]
#2	controlled clinical trial [pt]
#3	randomized [tiab]
#4	clinical trials as topic [mesh: noexp]
#5	randomly [tiab]
#6	trial [ti]
#7	OR/#1–#6
#8	Uremias [Mesh]
#9	Uremias [All Fields)
#10	OR/#8–#9
#11	Pruritis [Mesh]
#12	Itching OR Pruritis [All Fields)
#13	OR/#11–#12
#14	#10 AND #13
#15	Gabapentin [Mesh]
#16	1-(Aminomethyl)cyclohexaneacetic Acid OR Neurontin OR Gabapentin Hexal OR Convalis OR Gabapentin-Ratiopharm OR Gabapentin Ratiopharm OR Novo-Gabapentin OR Novo-Gabapentin OR Novo Gabapentin OR Novo Gabapentin OR NovoGabapentin OR PMS-Gabapentin OR Apo-Gabapenti OR Apo Gabapentin OR ApoGabapentin OR Gabapentin Stada [All Fields)
#17	OR/#15–#16
#18	Pregabalin [Mesh]
#19	(S)-3-(aminomethyl)-5-methylhexanoic acid OR 3-isobutyl GABA OR 3 isobutyl GABA OR 3-isobutyl OR 3-(aminomethyl)-5-methylhexanoic acid OR (R-)-3-isobutyl GABA OR (S+)-3-isobutyl GABA OR Lyrica OR CI 1008 OR 1008, CI OR CI-1008 OR CI1008 [All Fields)
#20	OR/#18–#19
#21	Tacrolimus [Mesh]
#22	Prograf OR Prograft OR FR-900506 OR FR 900506 OR FR900506 OR AnhydrousAnh Tacrolimus OR Tacrolimus, Anhydrous OR Tacrolimus ydrous OR Anhydrous, Tacrolimus OR FK-506 OR FK 506 OR FK506 [All Fields)
#23	OR/#21–#22
#24	Ondancetron [Mesh]
#25	Ondancetron [All Fields)
#26	OR/#24–#25
#27	#17 OR #20 OR #23 OR #26
#28	#7 AND #14 AND #27

### 2.5 Data collection

#### 2.5.1 Selection of studies

**Figure 1:**
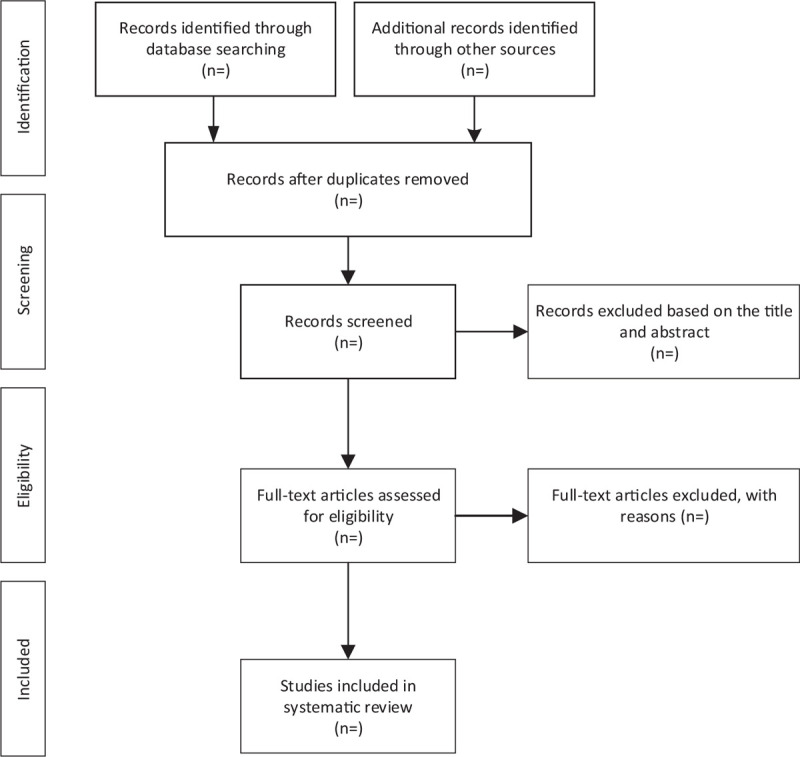
Flow diagram of study selection process.

For the convenience of management, we searched from five databases and imported titles into EndNote Software AQ8 (V.X9). First, we used the software to remove duplicate articles, then two team members (YT and YSF) independently read the titles and abstractions, they deleted the literature that did not meet the requirements, and read the full text of the remained articles to decide the final inclusion of the experiment. After that, cross-checking to the results of both parties was conducted. If there is any disagreement, the decision would be made via group discussion. The entire process and results are shown in Figure 1.

#### 2.5.2 Data extraction and management

We designed the information extraction table by using Microsoft Excel 2016, and carried out pre-extraction before the formal use, to ensure the feasibility of the table. Two trained members independently extracted the information in the article, including the following contents:

1. Basic information: Title author year language region diagnostic criteria
2. Baseline information: age and gender disease course sample size
3. Methodological quality information: the grouping method assigns the hidden blindness method result bias and other bias
4. Intervention measures: name of basic therapeutic drugs, dose-frequency usage, etc.
5. Results: Visual analogue Scale, VRS, DLQI (The Dermatology QOL Index) and adverse event incidence.

At the end of all information extraction, we will check the consistency of the two tables, and if there is any abnormal situation, we will determine the final result through group discussion.

### 2.6 Assessment of risk of bias in included studies

The two authors (YT and YSF) evaluated the article methodology of inclusive trials independently, by the Cochrane collaboration “Bias risk” tool sequences generated from six aspects of allocation concealment, blind (or mask), incomplete data evaluation, evaluation reports and other sources of bias selective results. Finally, for each items, we made ranking of “Low-risk bias,” “High-risk bias” and “Unclear” based on the Cochrane collaboration “bias risk” tool.^[19,20]^

### 2.7 Data analysis

#### 2.7.1 Management of lost data

When necessary data is missing in the included article, we will contact the author via email for complete data. If not, this article will be excluded. On the other hand, when the raw data is sufficient, we will calculate the required value according to the criteria in the Cochrane.^[21]^

#### 2.7.2 Network map

In the network diagram, each dot represents an intervention; the larger dot area means the bigger population of the studied intervention; the line between the two dots represents that there is direct comparison to RCT studies among two interventions; the line thickness represents the numbers of direct comparison to RCT studies among two interventions.

#### 2.7.3 Transitivity and consistency assessment

Transitivity and consistency are the prerequisites for reticular meta-analysis. The transitivity was evaluated qualitatively from the perspective of methodology and was evaluated according to the PICO principle. Consistency was mainly to check local and overall consistency. Local consistency can be checked by loop consistency test (Higgins model). The global consistency test was verified by the corresponding inconsistency model according to different data.

#### 2.7.4 Assessment of heterogeneity

Heterogeneity tests for all included studies were performed by using Network prediction interval graph, then to study the relationship of the weighted mean difference (WMD) at a 95% confidence interval (95% CI) and estimation zone (95%Prl) to invalid line, only when invalid line crosses perpendicularly to estimation zone but does not to CI, then means heterogeneity exists.^[22]^

#### 2.7.5 Pairwise meta-analysis

Two team members (YT and YSF) used statistical software—Stata (version 14.0, Stata Corporation, College Station, TX) for Pairwise meta-analysis. This is used for data analysis of direct comparisons, such as between two drugs with the same intervention in multiple RCTS.

#### 2.7.6 Network meta-analysis

Two team members (YT and YSF) used statistical software—Stata (version 14.0, Stata Corporation, College Station, TX) for analysis. A random effects model was used for network meta-analysis to compare the variables between different interventions. By comparing Surface Under the Cumulative Ranking Curve (SUCRA), the optimum intervention measures were determined. The range of SUCRA is 0% to 100%, the higher of the SUCRA means the better of the efficacy.^[23]^

#### 2.7.7 Assessment of reporting biases

The funnel plot will be used to verify publication bias. If the funnel plot is symmetric, there is no obvious publication bias; otherwise, there is publication bias.

#### 2.7.8 Subgroup analysis

In the heterogeneity test, if the result is positive, we will conduct subgroup analysis, group the included articles according to PICO principle, and use STATA 14.0 test in turn to determine the source of heterogeneity.

#### 2.7.9 Grading the quality of evidence

Two team members (YT and YSF) independently evaluate the evidence quality in four grades: “high,” “medium,” “low,” and “very low”; according to the standards in the Grading of Recommendations Assessment Development and Evaluation (GRADE) system.^[24]^ In the next step, the results will be exchanged for examination. If there are different opinions, a group discussion will be held to determine the conclusion.

### 2.8 Ethics and dissemination

This study is a secondary literature study and does not involve ethical issues. The results will be published in peer-reviewed journals designed to provide reviewed studies for the clinical treatment of uremic pruritus.

WMH conceived this study. YT and YSF completed the project, search strategy, research selection, bias risk assessment, data extraction, data analysis, and evidence quality assessment, LFS and HY assisted to the project revision and bias risk assessment, XL and LHQ assisted to the analysis and evidence quality assessment, YT wrote the original manuscript, YSF reviewed and edited the documents. All the authors approved the final project.

## Author contributions

**Conceptualization:** MaoHong Wang.

**Data curation:** Shifan Yan, Ting Yu, Lu Xiao.

**Formal analysis:** Shifan Yan, Ting Yu.

**Funding acquisition:** Yong Huang.

**Investigation:** Shifan Yan, Ting Yu.

**Methodology:** Shifan Yan, Ting Yu, Fusheng Li, Lu Xiao, Haiqun Li.

**Resources:** Yong Huang, MaoHong Wang.

**Supervision:** Fusheng Li, Lu Xiao, Haiqun Li.

**Validation:** Yong Huang, Fusheng Li, Haiqun Li.

**Writing – review & editing:** Shifan Yan.

**Writing – original draft:** Ting Yu.
